# Bone morphogenetic proteins, activins, and growth and differentiation factors in tumor immunology and immunotherapy resistance

**DOI:** 10.3389/fimmu.2022.1033642

**Published:** 2022-10-24

**Authors:** Shonik Ganjoo, Nahum Puebla-Osorio, Selene Nanez, Ethan Hsu, Tiffany Voss, Hampartsoum Barsoumian, Lisa K. Duong, James W. Welsh, Maria Angelica Cortez

**Affiliations:** Department of Radiation Oncology, The University of Texas MD Anderson Cancer Center, Houston, TX, United States

**Keywords:** TGF-β superfamily, bone morphogenetic proteins, activins, growth and differentiation factors, immunotherapy, cancer

## Abstract

The TGF-β superfamily is a group of secreted polypeptides with key roles in exerting and regulating a variety of physiologic effects, especially those related to cell signaling, growth, development, and differentiation. Although its central member, TGF-β, has been extensively reviewed, other members of the family—namely bone morphogenetic proteins (BMPs), activins, and growth and differentiation factors (GDFs)—have not been as thoroughly investigated. Moreover, although the specific roles of TGF-β signaling in cancer immunology and immunotherapy resistance have been extensively reported, little is known of the roles of BMPs, activins, and GDFs in these domains. This review focuses on how these superfamily members influence key immune cells in cancer progression and resistance to treatment.

## Introduction

The human transforming growth factor-β (TGF-β) superfamily is an extensive group of more than 33 polypeptides that regulate a multitude of vital developmental and homeostatic processes (1, 2). Dysregulation of TGF-β signaling has been implicated in several pathologic conditions, including cancer and autoimmune disorders (3–5). Specifically in the context of cancer, many previous studies have aimed to elucidate how the complex interplay between TGF-β signaling and key immune cells can either promote or inhibit a pro-proliferative phenotype (6–9). These studies have broadly established that TGF-β has context-dependent roles in mediating cancer immunity through pleiotropic effects (10–12). This context dependence and high variability in signaling have posed significant challenges for the development of TGF-β antagonists as effective cancer treatments (13, 14).

There are three known isoforms of TGF-β: TGF-β1, TGF-β2, and TGF-β3 (15). The immunoregulatory roles of these central members of the TGF-β superfamily have been extensively reviewed in the context of cancer; however, much less is understood about the roles of bone morphogenetic proteins (BMPs), activins, and growth and differentiation factors (GDFs), which are other key TGF-β superfamily members that immunologically affect cancer progression. BMPs, activins, and GDFs are highly conserved homo- or hetero-dimers with several morphologic similarities, but they can each exert distinct and potent effects on both innate and adaptive immune cells to modulate anti-tumor immunity (16–18). BMPs, activins, and GDFs are also similar to TGF-β in their having context-dependent effects on tumor progression; however, targeting certain ligands from these superfamily members has proven to be a viable treatment strategy in several cancer models (19–21). By extension, the signaling pathways initiated *via* type I and type II transmembrane receptors for BMP, activin, and GDF ligands also share many similarities with the canonical TGF-β signal transduction pathway. Studies have also demonstrated that either knockdown or overexpression of some of these receptors may be a promising treatment avenue for certain cancers (22, 23). Our group recently found that BMPs can promote resistance to immunotherapy by inhibiting the Th1 response in macrophages and T cells in a model of acquired resistance to PD1 therapy (24). Others have found that activins can promote immunosuppression by promoting regulatory T cells (T_regs_) in breast cancer (25).

In this Review, we explore the current understanding of how certain BMPs, activins, and GDFs individually influence cancer progression, tumorigenesis, and response to treatment. We further elucidate the mechanisms by which BMPs, activins, and GDFs regulate the activity of key innate and adaptive immune cells, thereby influencing tumor immunity. Finally, we examine how select BMPs, activins, and GDFs promote resistance to immunotherapy, opening avenues for further research into mechanisms of resistance as well as novel therapeutics.

## BMPs, activins, and GDFs: Ligands

### Bone morphogenetic proteins

BMPs are secreted growth factors with diverse functions in regulating developmental processes such as ectopic bone formation, embryogenesis, and neurogenesis (26–30). BMPs are highly conserved and morphologically similar ligands, but they are sub-categorized into at least four classes based on receptor specificity: BMP2/4, BMP5/6/7/8a/8b, BMP9/10, and BMP12/13/14 (26, 31, 32). In cancer, the role of BMPs is highly variable and context-dependent. For example, downregulation of BMP3 correlated with colorectal tumor progression, and re-introducing BMP3 in colorectal cancer cell lines significantly contributed to growth suppression (33). On the other hand, BMP2 has been found to stimulate the invasiveness of lung cancer cell lines *in vitro*, as well as markedly enhance lung tumor growth *in vivo (* 34). Moreover, conflicting studies have demonstrated that BMPs of a single type can have opposing effects on tumor progression (16). For instance, one study concluded that BMP4 strongly stimulates cell proliferation in pituitary prolactinoma models through SMAD/estrogen receptor crosstalk, implicating the involvement of downstream BMP4 signaling in promoting prolactinoma progression (35). In contrast, a separate study found that BMP4 inhibits breast cancer metastases by reducing granulocyte colony-stimulating factor expression and myeloid-derived suppressor cell activity (36). Evidently, no clear association exists between BMP signaling and tumorigenesis or metastases, but existing studies corroborate the contextually variable roles of BMP in cancer progression.

BMP7 is among the most widely investigated members of the BMP subfamily with respect to cancer, and studies across numerous cancer types have correlated BMP7 with poor prognosis and metastasis (24, 37–41). Moreover, the role of BMP7 in promoting resistance to various cancer therapies has opened new avenues for cancer treatment on a molecular basis. For instance, our group found that BMP7 promotes resistance to anti-PD1 therapy by inhibiting MAPK14 expression and inflammatory responses among macrophages and CD4^+^ T cells (24). Knocking down or neutralizing BMP7 and re-administering anti-PD1 therapy re-sensitized non-small cell lung tumor models to immunotherapy, presenting a novel treatment strategy for overcoming resistance to cancer immunotherapies (24). In addition to BMP7, a separate study also found that activation of the BMP2/4-BMPR signaling pathway conferred resistance to epidermal growth factor receptor tyrosine kinase inhibitors (EGFR-TKIs) in patients with lung squamous cell carcinoma harboring mutations in the EGFR gene (19). Combining EGFR-TKIs with inhibitors of the BMP receptor signaling pathway was also found to overcome resistance (19). Overall, BMPs are dynamic regulators of tumor progression and present key targets for overcoming resistance to cancer therapies in certain contexts.

### Activins

Activins are homo-dimeric or hetero-dimeric proteins consisting of two cross-linked β subunits (42). The three main bioactive activin dimers include activin A (βAβA), B (βBβB) and AB (βAβB) (17). Activins are generally involved in development and tissue homeostasis/repair, as evidenced by their roles in wound healing and scar formation (42–45). Activin A is the most widely studied member of the activin subfamily, although its role in cancer progression is not completely understood. Similar to BMPs, the role of activin A in tumor progression is context-dependent, in that its overexpression or inhibition can lead to increased proliferation based on cancer type (46–51). Although the effects of activin A signaling on unregulated cell growth are ambivalent, activin A has been found to predominantly increase the migratory propensity of various cells, promoting an invasive phenotype (52–54). Consequently, activin A has been implicated as a key mediator of metastasis in prostate and breast cancers, lung adenocarcinomas, and oral squamous cell carcinomas (55–58). On the other hand, several studies support the finding that activin A exerts anti-angiogenic effects by inhibiting the proliferation of vascular endothelial cells (59–61). Nevertheless, the competing pro-invasive effects of activin A seem to prevail over its anti-angiogenic effects, as melanoma cells overexpressing activin A still exhibited enhanced migration (62).

Activin A is also being investigated as a potential target for cancer therapy, although its paradoxical effects on tumor progression and morphologic similarity to other members of the TGF-β family have presented important challenges. For instance, in a phase I study, STM 434, an ActR-IIB–based ligand trap for activin A, resulted in stable disease in 53% of all patients and 80% of patients with granulosa cell ovarian cancer (20, 54). However, off-target interactions between STM 434 and BMP9 resulted in complications such as mucocutaneous bleeding (20). More specific methods of targeting activin A signaling (such as monoclonal antibodies and physiologic binding proteins) have not been directly explored in cancer as yet, but they have been investigated in muscular and other genetic diseases (63, 64). Because activin A has been found to promote cancer stemness and resistance to cancer therapies, it remains a desirable yet relatively elusive target for several cancer types (65–68).

### Growth and differentiation factors

GDFs are highly related to BMPs in overall morphology, and are in fact considered to be a part of the same cytokine lineage, with some redundancy in nomenclature (69). Like BMPs, GDFs also exist as dimeric proteins and regulate a variety of processes related to development, especially in the skeletal, muscular, and nervous systems (70–72). Most studies examining the role of GDFs in cancer have centered on GDF15 because of its being implicated in acute and chronic inflammatory diseases (73). In breast cancer, GDF15 has been found to enhance tumor proliferation and growth, potentially by increasing iron retention (18, 74). Several other studies have found GDF15 to contribute to the invasiveness and metastatic potential of breast cancer tumors through various signaling pathways, including p38 MAPK phosphorylation and EGFR transactivation (18, 75–78). Finally, GDF15 has been implicated in contributing to angiogenesis and the stemness of breast cancer tumors, largely through circuits that induce vascular endothelial growth factor expression and tumor sphere formation (76, 79). These pleiotropic effects of GDF15 in breast cancer (stimulating proliferation/growth, invasion, metastasis, angiogenesis, and stemness), are also applicable to other cancer types; indeed, GDF15 has been observed to contribute to tumor progression through one or more of these effects in gastric, pancreatic, colorectal, prostate, and cervical cancer, among several others (18, 21, 80–83). GDF15 has been established as a nexus for key hallmarks of cancer across numerous cancer types, and future work may entail exploring GDF15 signaling as a potential therapeutic target.

## BMPs and activins receptors

### BMP type I and II receptors

**Figure 1:**
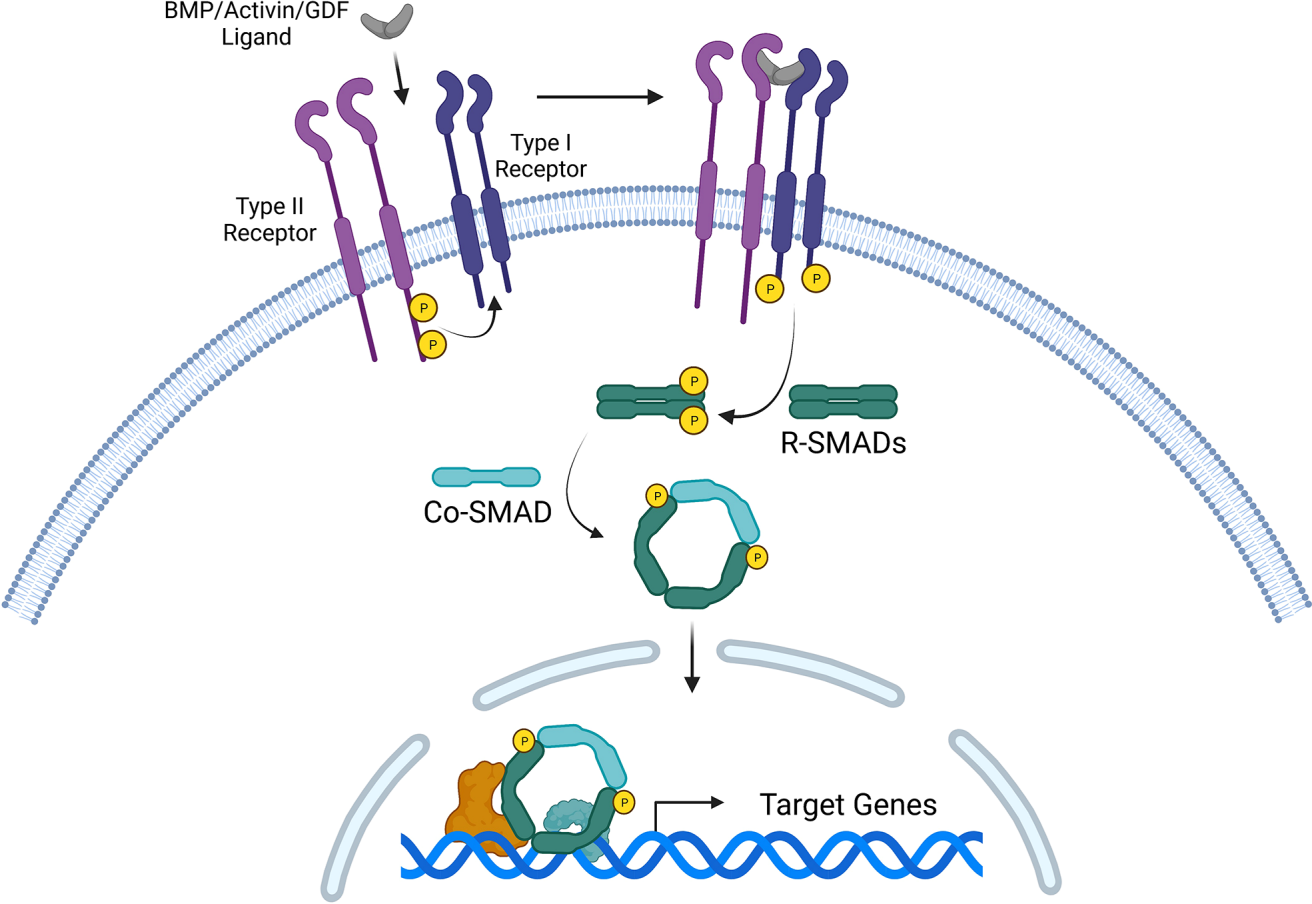
Canonical signaling pathway for three TGF-β superfamily members: bone morphogenetic proteins (BMPs), activins, and growth and differentiation factors (GDFs). Ligand binding induces the formation of a heteromeric receptor complex, resulting in constitutively active type II receptors transphosphorylating and activating type I receptors. The signal is transduced intracellularly *via* phosphorylated R-SMADs (such as R-SMAD1/5/8 or R-SMAD2/3). R-SMADs form complexes with Co-SMADs (such as SMAD4), which collectively serve as transcription factors that regulate target gene expression in the nucleus.

BMPs and GDFs signal through type I and type II receptors, both of which are serine-threonine kinase transmembrane receptors (84). BMPs and GDFs have highly specific functions, and homo- and hetero-dimers interact with combinations of type I and type II receptor dimers to produce numerous possible signaling complexes, leading to the activation of SMAD transcription factors (69). Although BMPs have been found to bind the type I receptor in the absence of the type II receptor, the presence of both receptors as a heteromeric complex significantly increases BMP binding affinity (84, 85). Three type II BMP receptors have been identified thus far: BMPR-II, ActR-II, and ActR-IIB; and four type I BMP receptors, characterized as part of the BMPR-I group (ALK1, ALK2, BMPR-IA/ALK3, BMPR-IB/ALK6) (86). Generally, BMP2 and BMP4 bind to BMPR-IA and BMPR-IB (87), BMP6 and BMP7 bind strongly to ALK2 and weakly to BMPR-IB, and BMP9 and BMP10 bind to ALK1 and ALK2 (88–90). GDF5 preferentially binds to BMPR-IB, but not to other type I receptors (91). However, different BMP dimers bind to different heteromeric receptor complexes with varying affinity depending on BMP type and receptor type (92–94). BMPR-II is specific for BMPs, whereas ActR-II and ActR-IIB are shared by activins, myostatin, and BMPs. These type II receptors seem to bind most BMP ligands and affect the binding preferences of BMPs to type I receptors (95). Although type I and type II receptors differ in intracellular structure and signaling, these receptors share a cysteine-rich extracellular domain that facilitates binding to BMP dimers (96). Type II BMP receptors are constitutively active and phosphorylate type I receptors, which subsequently initiate intracellular signaling, primarily through transphosphorylation of the receptor-regulated SMAD proteins (R-SMAD1, 5, and 8) (97). Activated R-SMADs can form heteromeric complexes with a common mediator, such as SMAD4, to regulate downstream transcriptional responses in the nucleus (86) (**Figure 1**).

BMP receptors also have context-dependent roles in tumor progression, with contrasting studies implicating BMP receptors as either stimulators or suppressors of tumor growth and metastasis. Mutations in genes encoding for ALK3 (BMPR-IA) have been observed in some patients with juvenile polyposis syndrome (84, 98, 99). BMPR-IA also inhibits squamocolumnar and gastrointestinal junction zones in mice, which are epithelial areas associated with enhanced oncogenesis (16, 100). However, deletion of BMPR-IA has also been found to impair mammary tumor formation and metastasis, suggesting that BMPR-IA may stimulate tumor progression in breast cancer (16, 22). Similarly, one study concluded that inhibition of BMPR-II expression inhibited chondrosarcoma tumor growth by inducing autophagy and apoptosis (101). In contrast, a separate study found that disrupting BMPR-II in mammary tumors stimulated tumor development and metastasis by promoting inflammation and infiltration of myeloid-derived suppressor cells (102).

Various small-molecule inhibitors of BMP receptors have presented as promising candidates for cancer therapy. For instance, dorsomorphin and LDN-193189, inhibitors of the type I BMP receptor ALK1, blocked cell migration and improved cell killing in models of epithelial ovarian cancers (103, 104). K02288, a small-molecule inhibitor that also targets ALK1, was found to inhibit BMP9 signaling as a critical step towards suppressing tumor angiogenesis in models of diffuse intrinsic pontine glioma (105). DMH1, another inhibitor of type I BMP receptors, was also found to reduce lung cancer cell proliferation, promote cell death, decrease invasion, and inhibit tumor growth in human lung cancer xenograft models by blocking BMP signaling (106). Collectively, BMP receptors are promising targets for cancer therapy, and future work may entail more thorough investigation of the mechanisms behind BMP receptor inhibition in suppressing tumor growth.

### Activin type I and II receptors

Activins, like BMPs, signal through transmembrane serine-threonine kinase type I and type II receptors that form heteromeric receptor complexes. The complexes requires two type I receptors (ALK4, 5, or 7) and two type II receptors (ActR-IIA or ActR-IIB) (107). The binding activity of these receptors is also similar to that of BMP receptor complexes: the type II receptor is primarily responsible for initial ligand binding and activation of the type I receptor, which can then propagate signaling through SMAD2 or SMAD3 (107). In activin signaling, SMAD2 or SMAD3 can form a complex with SMAD4 and co-localize to the nucleus, where the complex regulates various transcriptional responses (108). Activin receptors can also bind to ligands promiscuously. For instance, BMP10 binds to ActR-IIA and ActR-IIB (109). GDF11 and myostatin have also been shown to bind ALK4 and ALK5 (1, 108, 110). This functional redundancy in receptors across members of the TGF-β superfamily has important implications for the development of therapeutics, especially regarding potential off-target effects.

In cancer, the activity of activin receptors is largely context-dependent. In models of lung adenocarcinoma, ALK4 expression was associated with reduced survival by promoting resistance to platinum chemotherapy; inhibition of ALK4 improved response to chemotherapy and reduced chemotherapy-induced nephrotoxicity (67). Inhibition of ActR-IIB was found to reduce cancer-induced cachexia and prolong survival (111). On the other hand, overexpression of wild-type ALK4 was found to restore anti-proliferative effects of activin in pituitary tumor cells (23). Similarly, transfection of the prostate cancer cell line LNCaP with wild-type ALK5 restored the tumor suppressive effects of TGF-β, whereas loss of ALK5 activity has been linked with advanced disease stage and poor 4-year survival in patients with prostate cancer (112–114). In contrast, TGF-βRI kinase inhibitor II, an ALK5 inhibitor, was found to block the invasive phenotype of cancer cells in oral squamous cell carcinoma models (112, 115). Numerous small-molecule inhibitors of ALK4, 5, and 7 have also been shown to attenuate tumor progression and invasion in various cancer types, including osteosarcoma, breast cancer, glioblastoma, and hepatocellular carcinoma (112, 116–121). Among the most advanced targeting strategies deployed to interfere with TGF-β superfamily signaling in cancer is coupling the small molecule inhibitor galunisertib with anti-PD1 immunotherapy, which resulted in complete regression in murine colon cancer models (54, 122). However, whether galunisertib targets the activin A receptor ActRIB, the TGF-β receptor TGFBRI, or both with differential affinity remains unclear. Overall, targeting activin receptors as a means of treating cancer has proven to be a viable strategy based on outcomes from several clinical studies, but as is true for BMP receptors, a deeper understanding of the mechanism behind activin receptor inhibition can further expand therapeutic avenues that use this approach.

## Interactions of BMPs, activins, and GDFs with the innate immune system in cancer

### Bone morphogenetic proteins

**Figure 2:**
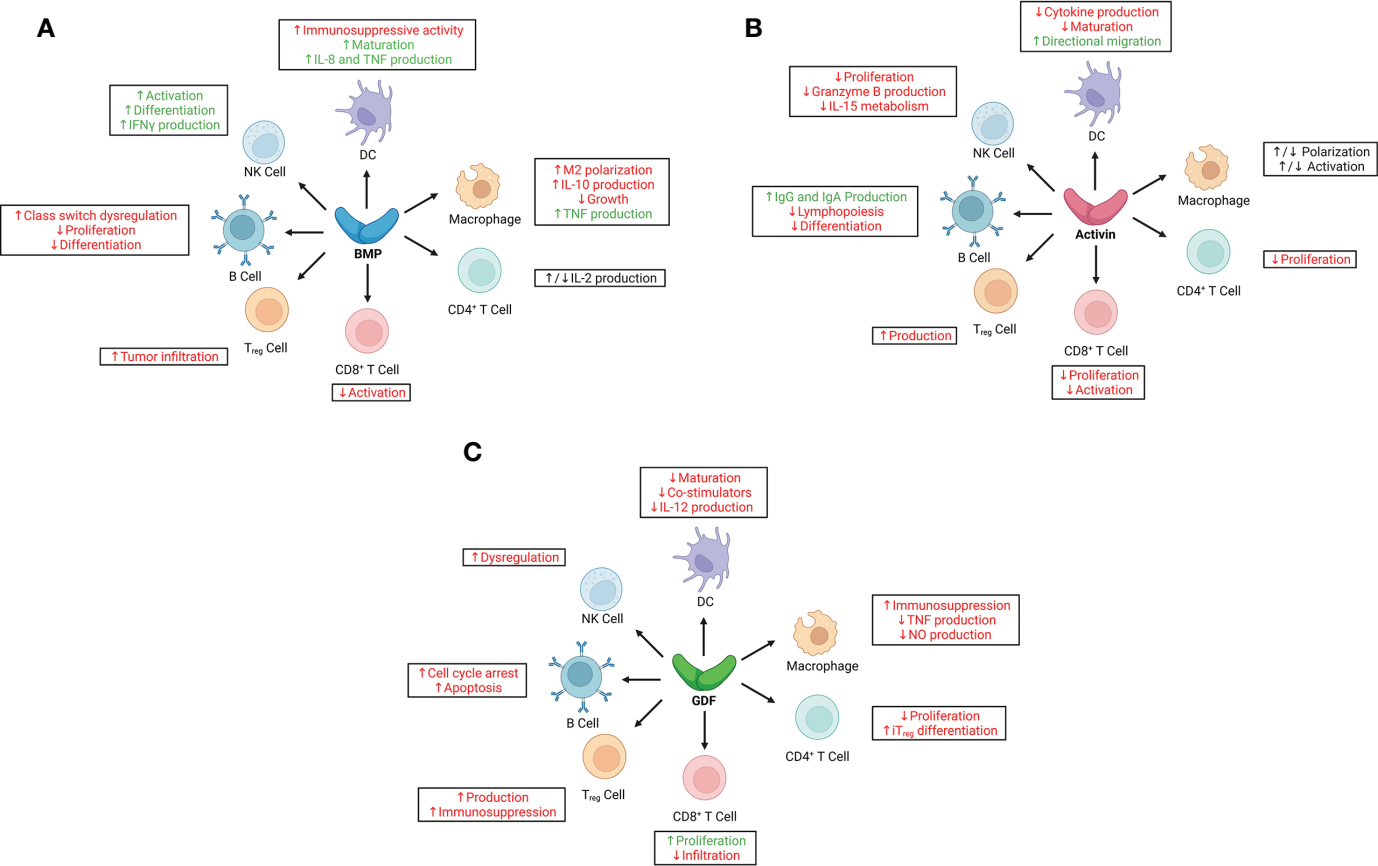
Immunoregulatory effects of bone morphogenetic proteins (BMPs), activins, and growth and differentiation factors (GDFs) on key cells of the innate and adaptive immune systems. **(A)** BMPs have largely pro-inflammatory effects in adaptive and innate immune responses, although their immunoregulatory roles in cancer are context-dependent, especially with regard to signaling in natural killer (NK) cells, macrophages, and CD4^+^ T cells. **(B)** Activins have predominantly anti-inflammatory effects in adaptive and innate immune responses; however, like BMPs, their immunoregulatory function in cancer is largely context-dependent, as evidenced by paradoxical signaling to macrophages and some dendritic cells (DCs). **(C)** GDFs have broad immunosuppressive effects on both adaptive and innate immune responses, correlating with poor outcomes in various cancer types. Red text indicates pro-tumorigenic effects; green, anti-tumorigenic effects. IFNγ, interferon-γ; IL, interleukin; iT_reg_, induced regulatory T cell; NO, nitric oxide; TNF, tumor necrosis factor; T_reg_, regulatory T cell.

In the context of oncology, BMPs have a role in cancer development, progression, and immune regulation. They can modulate dendritic cells (DCs) that express type-I receptors (BMPR-IA and ALK2) and BMPR-II receptors (123). BMPs are also involved in macrophage and natural killer (NK) cell activity (**Figure 2**).

BMPs promote tumor progression in breast cancer and promote metastasis to the bone (124). Overexpression of BMP2, for example, correlated with poor survival outcomes in ovarian cancer; BMP2 enhanced the migration and invasion of ovarian cancer cells, suggesting that BMP signaling promotes tumor progression and metastasis (125). In hepatocellular carcinoma, expression of BMP4 and BMP7 was increased, but advanced non-small cell lung cancer was associated with increased BMP2 serum levels and correlated with poor outcomes (104). BMPs are capable of inhibiting cancer cell proliferation in a context-dependent manner; however, they simultaneously promote cancer cell invasion. BMPs promote tumor growth by inhibiting the function of DCs, which express BMP ligands and receptors, driving M2-like macrophage development, and upregulating PDL1 and PDL2; however, in a paradoxical role, they can promote NK cell activity, differentiation, and production of interferon (IFN) -γ (12, 126, 127).

In acute lymphoblastic leukemia, DC differentiation was altered into an aberrant phenotype displaying immune suppressive functions (128). In addition to DCs, macrophage differentiation was also affected so as to secrete and overexpress BMP4, which induces DCs with immunosuppressive functions, skews M1 macrophage polarization, and generates tumor promoting M2-like macrophages (128). However, separate studies have concluded that BMPs promote DC maturation and enhance their production of interleukin (IL) -8 and tumor necrosis factor (TNF) (123). BMP7 also influences macrophage M1 polarization to M2 (129). Tumor cells secrete BMP7 and act on macrophages in the tumor microenvironment and impair pro-inflammatory responses (24). In the human acute monocytic leukemia cell line THP-1, BMPR-II and BMP7 polarize monocytes to M2 macrophages (130). Tumor-associated macrophages also were found to secrete BMP2 in the tumor microenvironment in breast cancer, which led to poor prognosis (131). BMP6 was also found to inhibit the growth of macrophages and induce macrophages to produce IL-10, thereby suppressing an anti-tumor immune response. Paradoxically, BMP6 was also found to promote TNF production by macrophages (132, 133). Thus although BMPs generally suppress a variety of anti-tumor responses by innate immune cells, the mechanisms by which BMPs regulate the activities of these cells, often paradoxically, are not fully understood and warrant further research.

### Activins

Activin A, another member of the TGF-β superfamily of growth and differentiation factors, has several physiologic roles. It has both pro-inflammatory and anti-inflammatory properties, and is essential in the apoptosis of tumor and immune cells, wound healing, and cancer (134). In one study, activin A was shown to impair NK cell proliferation and inhibit production of granzyme B, which blunted tumor killing (135). When activin A binds to ALK4 on NK cells, SMAD2 and SMAD3 are phosphorylated, suppressing IL-15–mediated NK cell metabolism (135). Inhibiting activin A also has regulatory effects on DCs at different times. Activin A induces the directional migration of immature myeloid DCs (136). However, when endogenous activin A was inhibited by follistatin *in vitro*, CD40L stimulation upregulated the expression of numerous cytokines (IL-6, IL-8, IL-10, IL12, and TNF-α) by DCs and promoted the maturation of those cells (137). Moreover, other studies have shown that activin A enhances pro-inflammatory mediators (IL-1β, nitric oxide, and prostanoids), increases phagocytic activity, and skews macrophages to the pro-inflammatory macrophage phenotype (M1) (138). Activin A was shown to inhibit M2 macrophage genes consisting of C-maf proto-oncogene, insulin-like growth factor 1, and plasminogen activator inhibitor 2. When activin A was inhibited, M2 macrophage polarization increased (139). Contrarily, other studies proposed that activin A had a more suppressive role, and its secretion by T_H_2 cells promoted M2-like macrophage polarization (140). The multipronged effects of activin A on macrophage activity warrant further clarification of its role in mediating signaling pathways.

### Growth and differentiation factors

GDF15 has served as a biomarker for cancer tumorigenesis, prognosis, and progression (18). Its overexpression has been linked with poorer outcomes in various types of cancer. GDF15 was recently shown to have immune regulatory functions and has potential for immunotherapy targeting strategies (141). GDF15 polarizes macrophages in the tumor microenvironment into an immunosuppressive state by inhibiting TAK1 signaling to NF-kB and blocking production of TNF and nitric oxide (142). When GDF15 was depleted *in vivo* in a pancreatic cancer model with a Ras-driven tumor, immune surveillance was restored and tumor development was delayed, suggesting improved tumor control (142). Another recent study showed that GDF15 suppresses the maturation of DCs as well as their costimulatory molecules (CD83, CD86, HLA-DR). GDF15 enhanced phagocytosis by reducing the maturation of DCs. DCs treated with GDF15 secreted less IL-12 and more TGF-β1 inhibitory cytokine. Also, T-cell stimulation and cytotoxic T cell (CTL) activation by DCs was inhibited with GDF15 (143). The roles of GDF15 in the context of NK cells are cancer is not well understood; however, GDF15 has been linked with NK cell dysfunction through TGF-βR1 (144).

## Interactions of BMPs, activins, and GDFs with the adaptive immune system in cancer

### Bone morphogenetic proteins

BMPs, activins, GDFs, and their receptors are expressed in several arms of the immune system, and several studies have elucidated the roles of these ligands in adaptive immunity (12). Intra-thymic CD34^+^ cells express BMP receptors (BMPR-IA, BMPR-IB, ActR-IA, BMPR-II), signal transduction molecules (SMAD1, 5, 8 and 4), and produce BMP4. The role of BMPs on T cells was first described in the ontogenesis of T lymphocytes and thymic development, in which cells of the developing thymic stroma secrete BMP2 and BMP4, and immature double-negative (CD4^–^, CD8^–^) thymocytes express BMP receptors (145). BMP has been found to be essential in early lymphocyte differentiation, as inhibition of BMP (146) or the conditional deletion (147) of BMPR-IA in early development results in reduced thymocyte populations and a smaller thymus. Also, BMP4 mediates the epithelial-mesenchymal interactions during thymic development and parathyroid morphogenesis (148), and interacts with IL-7 in the maintenance of the human thymic progenitor population (149).

Human naïve CD4^+^ T cells express transcripts for BMPR-IA, ALK2, and T cell receptors (TCRs). Stimulation of those cells by IL-7 results in activation of the BMP pathway, with essential effects on their homing receptor expression, survival, and homeostatic proliferation (150). Inactivation of BMPR-IA in T cells impairs the thymic and peripheral generation of T_regs_, and BMPR-IA-deficient, activated T cells increase IFN-γ production. *In vivo* studies demonstrated that conditional deletion of BMPR-IA in T cells results in a more effective anti-tumor immune response, a higher proportion of activated anti-tumor CD8^+^ cells, and fewer infiltrating tumor-infiltrating T_regs_ (151). Contrasting evidence shows that inhibiting BMP *in vitro* leads to reduced expression of IL-2Ra and IL-2 secretion in CD4^+^ T cells (152, 153), yet BMP7-treated, activated CD4^+^ T cells reduced their secretion of IL-2 (24). Such opposing findings indicate that elucidating the role of BMP in lymphocyte function is far from complete. Further, impairing BMP signaling with dorsomorphin suppressed phosphorylated SMADs 1/5/8 in peripheral CD4^+^ T cells. Dorsomorphin induced cell cycle arrest in T cells at G1, suppressed Th17 cells, and promoted the differentiation of T_regs_. The inhibition of BMP also suppressed IL-2 production in mouse CD4^+^ T cells, suggesting that BMP-SMAD signaling physiologically regulates IL-2 transcription in CD4^+^ T cells (153).

### Activins

In an *in vivo* model of autoimmune encephalomyelitis, mice receiving activin A showed a significant improvement in encephalomyelitis, with decreased numbers of IFN-γ^–^, IL-17^–^, and granulocyte-macrophage colony-stimulating factor^–^producing CD4^+^ T cells, as well as an increase in IL-10-producing cells. Activin A treatment also impaired Th17 pathogenicity *via* CD39 and CD73 ectonucleotidase responses (154).

Activin A induces CXCR5 and PD1 expression and regulates the differentiation of CD4^+^ follicular helper T cells (TFH), functions of which include promoting survival, affinity maturation, and class switch recombination of B cells in the lymph node and the spleen. IL-2 has been shown to impair the role of activin A in the SMAD2/3-dependent programming of TFH (155).

*In vitro* irradiation of mouse and human breast cancer cells leads to significant increases in activin A expression. *In vivo*, radiation-induced activin A led to an increase in T_regs_ (25) and an increase in TGF-β levels (156). Conversely, blocking activin A and TGF-β reduced the levels of T_regs_ and promoted CD8^+^ T cells proliferation and tumor control. Thus, targeting activin A may have therapeutic relevance for minimizing the radiation-induced T_reg_ population (25). Activin A has inhibitory effects on antigen-specific Th2 and Th1 responses *via* the induction of T_regs_. Blocking IL-10 and TGF-β1 reverses the inhibitory effects of activin A (156). Activin A has also been shown to replicate the function of TGF-β1, driving the generation of IL-21-producing Th9 cells, which has implications for exacerbated allergic conditions (157). Importantly, Activin A is an important contributor to immune suppression and a regulator of inflammation through the conversion of CD4^+^/CD25^–^ T cells into induced CD4^+^/CD25^+^/FoxP3^+^ T cells in synergy with TGF-β1 (158).

GDF15 is a critical regulator of T-cell activity, and its role in immune modulation has been elucidated in several studies. Downregulation of GDF15 was found to improve T-cell infiltration into transplantable glioblastoma, prolonging survival and enhancing the immune response (159). GDF15 was also found to promote immunosuppression by enhancing the generation of T_regs_ in hepatocellular carcinoma (141). Specifically, GDF15 led to decreased proliferation of naïve CD4^+^ T cells while effectively promoting their differentiation into inducible T_regs_. However, GDF15 has also been found to stimulate tumor immunity, as its overexpression was correlated with increased populations of activated CD8^+^ T cells in murine models of prostate cancer (160).

The BMPR-IA signaling pathway regulates differentiation and self-renewal in several stem-cell populations in the germinal center. Mouse germinal-center B cells showed increased expression of BMPR-IA, and the targeted deletion of BMPR-IA impaired the germinal-center reaction and reduced differentiation from plasmablasts to antibody-producing plasma cells (161). One study identified that although human germinal-center B cells express high levels of BMPRI and low levels of BMPRII, naïve B cells show low levels of BMPRI and high levels of BMPRII (162). In the same study, BMP7 was shown to negatively regulate the survival of germinal-center B cells, and the truncated form of TGFβ-R1 reversed that effect.

The exogenous exposure of both normal donor naïve CD27^–^ and memory CD27^+^ B cells to BMP2, 4, 6, and 7 was found to prevent the CD40L/IL-21 stimulation of immunoglobulins (Ig) M, IgG, and IgA, suggestive of a strong dysregulation of class switch recombination and suppression of memory B cells (163).

Activin A has an inhibitory role in the generation of B-cell lineages in the bone marrow, and researchers argue that activin A may have a morphogen-like role during hematopoiesis (164). Moreover, activin A produced by mesenchymal stromal cells was found to negatively control B-cell lymphopoiesis, affecting B-cell lineage production (165). Activin A has also shown effects on class switch recombination in murine B cells, in that activin A increased the secretion of IgA by mesenteric lymph-node B lymphocytes independent of TGF-β (166).

### Growth and differentiation factors

*In vitro* studies suggest that GDF5 may be involved in signaling of the B-cell lineage; because GDF5 induced G1 cell-cycle arrest in mouse B-cell hybridoma HS-72 cells; the ectopic expression of SMAD6 and 7 reversed this effect *via* suppression of p21 and dephosphorylation of the Rb protein. The investigators argued that the potential inhibitory effect of SMAD6 and 7 on GDF5 could mediate the fate of B-lineage cells (167).

## BMPs, activins, and GDFs in resistance to immunotherapy

Our group looked at the effects of BMP7 in an anti-PD1 tumor model involving a variant of a murine lung cancer cell line. We found that BMP7 contributed to immunotherapy resistance by reducing proinflammatory signaling *via* suppression of MAPK14 (24). Conversely, when BMP7 was neutralized or knocked down, anti-PD1 non-small cell lung cancer tumors were re-sensitized to immunotherapy (24). Another group found that BMP2/4-BMPR-SMAD1/570S6K activation promoted resistance to erlotinib, an EGFR-TKI, in lung squamous cell carcinomas with EGFR mutations (19).

Activin A has also been shown to confer resistance to immunotherapy in mammary carcinoma by promoting T_regs_ (25). Secretion of activin A by radiation-induced breast cancer cells contributed to an increase in T_regs_, leading to increased resistance to immunotherapy in a mouse model (25). Activin A knock-ins in the low-activin-A-secreting breast cancer cell line TSA were also shown to hinder abscopal anti-tumor immune responses to radiation therapy in mice (25). Finally, to further assess how activin A influences immunotherapy resistance, blockade of both activin A and TGF-β led to an increase in survival rate and eliminated irradiated tumors in 57% of irradiated mice (25). Another group found that activin A secreted by melanoma inhibited CD8^+^ T cell immunity, ultimately promoting tumor resistance (168). This resistance to immunotherapy led to increased cachexia and tumor angiogenesis and resulted in poorer prognosis (168). Activin A secreted from melanoma cells also hindered proinflammatory signaling of cytokines and chemokines and shifted the composition of tumor immune infiltrates in the tumor microenvironment from CTLs and NK to increased non-regulatory cells such as CD4^+^ T cells, DCs, and monocytes.

Some evidence also exists to suggest that GDFs can promote immunotherapy resistance. In one study of hepatocellular carcinoma cells, GDF15 was found to increase T_regs_ mediated by CD48, leading to immunosuppression. In contrast, GDF15 knockout in tumor cells inoculated in mice led to decreased proportions of T_regs_, reduced tumor growth, and prolonged survival. These findings demonstrate that GDF15 can contribute to immunotherapy resistance by promoting the generation of T_regs_. Consequently, GDF15 blockade resulted in hepatocellular carcinoma clearance through an enhanced antitumor immune response (141).

In summary, although the mechanisms underlying resistance to immunotherapy remain unclear, BMPs, activins, and GDFs seem to confer resistance to treatment in a context-dependent manner (24, 25, 141). Little research has been reported to date that addresses this process, and additional research is needed to further elucidate immunotherapy resistance. Understanding this phenomenon could lead to the development of novel methods to combat resistance to immunotherapy.

## Clinical potential for targeting BMPs, activins, and GDFs in immunotherapy

Although research on targeting BMPs, activins, and/or GDFs to improve responses to cancer immunotherapy is still in nascent stages, early evidence affirms that these TGF-β superfamily members are promising candidates on a clinical basis. For instance, DMH1, a second-generation dorsomorphin analog, is a highly selective inhibitor of Type I BMP receptors. Mouse models of breast cancer treated with DMH1 demonstrated anti-metastatic function and significantly increased infiltration of immune cells compared to controls (102, 169). Consequently, these findings raised the exciting prospect that targeting BMP could enhance the effectiveness of immunotherapy based on the total number of myeloid and CD45^+^ immune cells present within the tumor (170).

Another study found that removing the *BMPR1α* gene in T-cells at the double-positive stage resulted in lower levels of T_regs_ and more IFNγ-producing T-cell subpopulations. These findings have important implications for potentially overcoming a major obstacle to effective cancer immunotherapy: infiltration of FoxP3^+^ T_regs_ that mediate immune tolerance (171). *BMPR1α*-deficient mice also demonstrated slower growth rates of melanoma tumors, bolstering the conclusion that removal of Type I BMP receptors could enhance an immune response to cancer (151). Additionally, we found that knockdown or neutralization of BMP7 re-sensitized resistant tumors to anti-PD1 immunotherapy in pre-clinical models (24). Altogether, early success in inhibiting BMPs and/or their receptors across these studies reinforce the clinical potential of targeting BMPs to improve responses to immunotherapy in specific cancers.

A recent study also found that activin A expression is positively correlated with anti-PD1 therapy resistance in human melanoma patients based on a bioinformatics analysis of available RNA-seq datasets. In the same study examining pre-clinical melanoma models, expression of the activin A-encoding gene, *INHBA*, was shown to reduce infiltration of cytotoxic T-lymphocytes and natural killer cells across all models. Additionally, activin A was responsible for reprogramming the tumor microenvironment to interfere with proper activation of cytotoxic T-lymphocytes and attenuated levels of IFNγ in bulk tumors. Notably, neutralization of endogenous activin A by a soluble form of ActR-IIB sensitized melanoma grafts to combined anti-PD1/anti-CTLA4 blockade, supporting the clinical potential of targeting activin A to improve responses to immunotherapy in melanoma (168).

GDF15 is also an emerging target for improving responses to immunotherapy, particularly because it is a biomarker correlated with immune-neglected “cold” tumors, which are significantly less responsive to immune checkpoint blockade (159, 172, 173). One study aiming to explore the immunologic role of GDF15 in glioblastoma models revealed that GDF15 promotes the expression of PD-L1 through activation of the SMAD2/3 signaling pathway. The results from this study suggest that targeting the GDF15/PD-L1 pathway could be an effective immunotherapy strategy to enhance anti-tumor immunity in glioblastoma (174). In other models of cancer that respond poorly to immunotherapy, such as hepatocellular carcinoma (HCC), GDF15 is also an ideal target because it plays a central role in the generation and activation of T_regs_, thereby mediating an immunosuppressive response (141). In mice models, therapeutic blockade of GDF15 was found to achieve clearance of HCC when coupled with anti-PD1 mAb treatment (141).

Early research continues to provide promising results for targeting BMPs, activins, and GDFs to increase the effectiveness of immunotherapy in treating certain cancers. The previously discussed studies support the immunosuppressive effects of these TGF-β superfamily members and have used these effects to inform the development of combined therapies that were successful in bolstering anti-tumor immune responses. However, further research is still required to investigate additional parameters such as off-target effects, refractory immunotherapy resistance, and cancer-specific/context-dependent effects of inhibiting these superfamily members to validate the clinical safety and efficacy of targeting them.

## Author contributions

The concept of this review was developed by MC and SG. SG: writing–review and editing, NP-O: writing–review, SN: writing–review, EH: writing–review, TV: writing–review, HB: writing–review, LD: writing–review, JW: writing–review, and MC: writing–review and editing. All authors contributed to the article and approved the submitted version.

## Funding

This work was supported U.S. Department of Defense (DOD) Lung Cancer Research Program (LRP; W81XWH-21-1-0336) to MC.

## Acknowledgments

The authors thank Christine F. Wogan for providing editing support during the preparation of this manuscript.

## Conflict of interest

The authors declare that the research was conducted in the absence of any commercial or financial relationships that could be construed as a potential conflict of interest.

## Publisher’s note

All claims expressed in this article are solely those of the authors and do not necessarily represent those of their affiliated organizations, or those of the publisher, the editors and the reviewers. Any product that may be evaluated in this article, or claim that may be made by its manufacturer, is not guaranteed or endorsed by the publisher.
